# The *hAT*-family transposable element, *hopper*, from *Bactrocera dorsalis* is a functional vector for insect germline transformation

**DOI:** 10.1186/s12863-020-00942-3

**Published:** 2020-12-18

**Authors:** Alfred M. Handler, Marc F. Schetelig

**Affiliations:** 1 USDA/ARS, Center for Medical, Agricultural and Veterinary Entomology, 1700 SW 23rd Drive, Gainesville, FL 32608 USA; 2 Department of Insect Biotechnology in Plant Protection, Justus-Liebig University Gießen, Winchesterstr. 2, 35394 Gießen, Germany

**Keywords:** Insect genetic modification, Transposon-mediated transformation, Tephritidae

## Abstract

**Background:**

The *hopper hAT*-family transposable element isolated from the Oriental fruit fly, *Bactrocera dorsalis*, is distantly related to both the *Drosophila hobo* element and the *Activator* element from maize. The original 3120 bp *hopper*^Bd-Kah^ element isolated from the Kahuku wild-type strain was highly degenerate and appeared to have a mutated transposase and terminal sequences, while a second 3131 bp element, *hopper*^Bd-we^, isolated from a *white eye* mutant strain had an intact transposase reading frame and terminal sequences consistent with function.

**Results:**

The *hopper*^Bd-we^ element was tested for function by its ability to mediate germline transformation in two dipteran species other than *B. dorsalis*. This was achieved by creating a binary vector/helper transformation system by linking the *hopper*^Bd-we^ transposase reading frame to a *D. melanogaster hsp70* promoter for a heat-inducible transposase helper plasmid, and creating vectors marked with the *D. melanogaster* mini-*white*^+^ or *polyubiquitin*-regulated DsRed fluorescent protein markers.

**Conclusions:**

Both vectors were successfully used to transform *D. melanogaster*, and the DsRed vector was also used to transform the Caribbean fruit fly, *Anastrepha suspensa*, indicating a wide range of *hopper* function in dipteran species and, potentially, non-dipteran species. This vector provides a new tool for insect genetic modification for both functional genomic analysis and the control of insect populations.

## Background

Transposon-mediated germline transformation has been the primary method of insect genomic manipulation since a *P* element vector was successfully transposed into the *Drosophila melanogaster* genome [1]. More recent gene-editing techniques, such as CRISPR/Cas9, have provided additional methods for genome manipulation, but thus far are limited in achieving genomic integration of DNA constructs greater than several kilobases [2]. This limitation is especially critical for the development of genetically modified strains to improve biologically-based strategies for the control of insect populations harmful to agriculture and human health, for which conditional lethal and other transgene constructs are typically 10 kb or greater. Significantly, gene-editing is also limited in generating random genomic insertions for mutagenesis and enhancer-trap screens that have proven highly advantageous for functional genomic analysis, most clearly demonstrated by elucidating gene function and regulation in the *D. melanogaster* model system. Thus, the ability to use transposon vector systems such as the *P* element in a wide range of insect systems has been, and remains, a high priority for understanding and manipulating insect genomes.

Unfortunately, *P* element mobility was found to be atypically limited to the *Drosophila* genus and closely related species and has never been successfully used to achieve transposon-mediated transformation in a non-drosophilid species [3]. More than a decade passed before other Class II transposable elements were discovered that were functionally less restricted and successful in achieving non-drosophilid transformation, which included *mariner*/*Mos*1 discovered in *D. mauritiana* that initially transformed *Aedes aegypti* [4, 5], *Minos* discovered in *D. hydei* that initially transformed *Ceratitis capitata* [6, 7], *piggyBac* from the cabbage looper moth, *Trichoplusia ni*, that also initially transformed *C. capitata* [8, 9], and the *Hermes* element found in *Musca domestica* [10] used initially to transform *Ae. aegypti* [11]. *Hermes* is a member of the *hobo, Activator, Tam3* (*hAT*) family of transposons that has been found to be phylogenetically widespread [12]. While this supports the notion of widespread functionality, most full-length *hAT* elements (having a coding region and at least one terminal repeat sequence) were found to be defective, and thus far none, other than *Hermes*, have been shown to support insect transformation.

One of the defective *hAT* elements, *hopper*^Bd-Kah^, was originally discovered by use of a short *hAT*-related PCR product as a hybridization probe to a lambda GEM12 genome library of the Kahuku wild type strain of the Oriental fruit fly, *Bactrocera dorsalis* [13]. The sequenced element was found to be 3120 bp in length with 19 bp terminal inverted repeat (TIR) sequences with a single mismatch. The TIRs surrounded a 1.9 kb consensus *hAT* transposase transcriptional unit that was, however, interrupted by two frameshift mutations consistent with it being non-functional, in addition to a degenerate 8-bp genomic insertion site duplication. Southern blots to genomic DNA from several *B. dorsalis* strains, and those from the melon fly, *Zeugodacus cucurbitae*, showed the presence of elements highly conserved with *hopper*^Bd-Kah^ indicating the existence of related elements in, and possibly beyond, the genus.

In an effort to discover a functional paralog of *hopper*^Bd-Kah^, 5′ and 3′ primers to the *hAT* element were used in opposite orientation for inverse PCR in one of the other *B. dorsalis* strains carrying a *white eye* (*we*) mutation and the presence of elements similar to *hopper*^Bd-Kah^ [14, 15]. This led to the discovery of a new 3131 bp element in *B. dorsalis*, *hopper*^Bd-we^. Unlike *hopper*^Bd-Kah^, *hopper*^Bd-we^ was found to have an uninterrupted 1950 bp reading frame, intact 19-bp TIR and 16-bp sub-TIR sequences, and a duplicated 8-bp insertion site sequence consistent with a functional transposon. While this full-length element provided insights into the conserved functional domains for Class II elements in general, and *hAT* elements specifically, its ability to autonomously transpose (requiring functional transposase and requisite terminal sequences) remained unknown. Function for *hopper*^Bd-we^ was implied by preliminary embryonic transposon mobility assays in *B. dorsalis*, however, function for the *D. melanogaster hobo hAT* element was also implied for five tephritid species using simlar assays [16], yet in our hands it was never successful in achieving non-drosophilid germline transformation. Thus, we initiated more direct and conclusive tests for *hopper*^Bd-we^ function based on its ability to mediate in vivo germline transformation in two dipteran species, *D. melanogaster* and *A. suspensa*.

## Results

### Transformation experiments

**Fig. 1:**
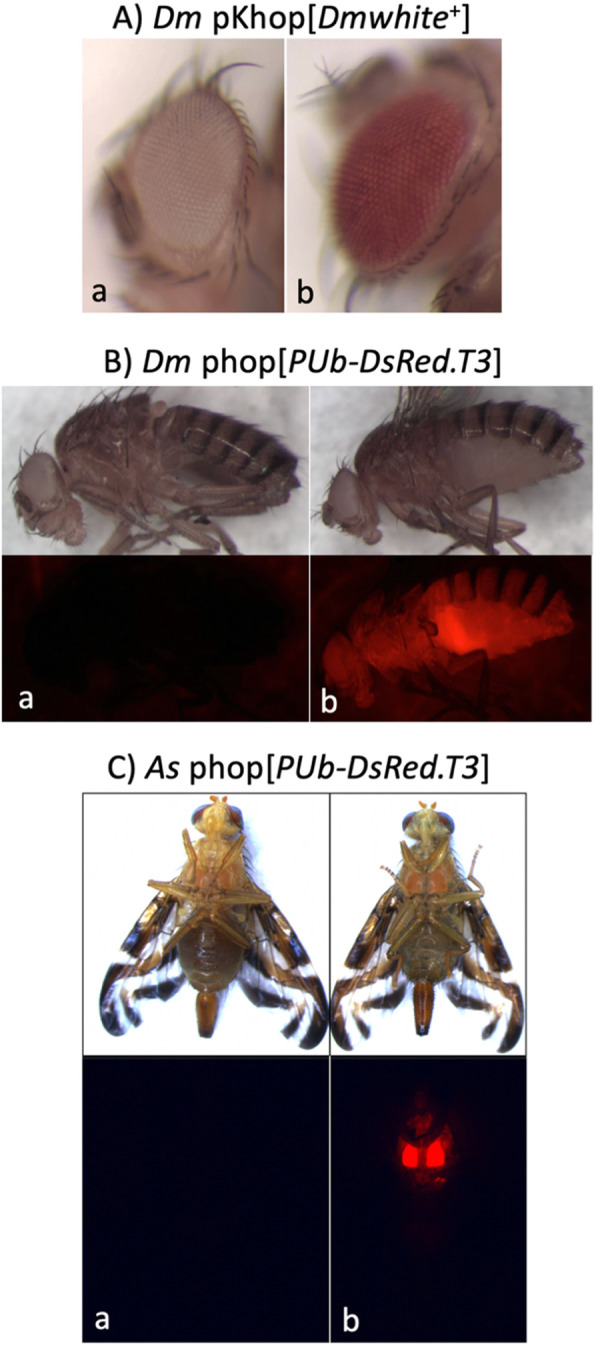
Phenotypes of *D. melanogaster* (*Dm*) and *A. suspensa* (*As*) transformed with the pKhop[*Dmwhite*^+^] (A) or phop[*PUbDsRed.T3*] (B, C) *hopper* transposon vectors. Panel Aa shows the *D. melanogaster w*[m] host strain *white* eye phenotype and Ab shows the red pigmented eye mutant-rescue phenotype after pKhop[*Dmwhite*^+^] transformation under brightfield. Panels B and C show host strain individuals (Ba and Ca) and transformed individuals (Bb and Cb) under brightfield (top) and Texas Red epifluorescence (bottom)

In the first of three transformation experiments we tested the *hopper*^Bd-we^ transposon vector system (now referred to as *hopper*) in the *D. melanogaster w*[m] *white* (eye) mutant host strain using the *hopper* vector, pKhop[*Dmwhite*^+^], marked with the *D. melanogaster* wild type mini-*white*^+^ gene. This serves as a transformant mutant-rescue marker by complementing the host strain *white*^−^ mutation, resulting in eye pigmentation in G_1_ germline transformants. The marker insertion within the transposase coding region creates a non-autonomous vector that relies on an exogenous source of transposase for transposition, that was provided by the pUChsHopper helper plasmid having the transposase gene under *D. melanogaster hsp70* promoter regulation for induction of expression by heat shock [17]. A mixture of vector and helper plasmids was injected into 436 dechorionated eggs under halocarbon oil, from which 131 G_0_ adults survived (Table 1). A total of 70 adults were backcrossed individually to *w*[m] adults, with an additional 61 G_0_ adults mated in 28 groups of 2 to 3 to *w*[m] adults of the opposite sex, for a total of 98 G_0_ lines. Seventy-nine G_0_ fertile matings were screened for G_1_ progeny with pigmented eyes that were observed in two of the matings, F11A and F23A (Fig. 1a), resulting in a transformation frequency of 2.5% per fertile G_0_. The F11A G_0_ mating yielded three transformant progeny that were considered to be G_1_ siblings resulting from the same transformation event. It is possible for marked G_1_ siblings to arise from independent vector insertions, however all three lines shared the same female-specific marker phenotype that was mapped to the X-chromosome in F11A, consistent with a common germline transformation event (see below).

**Table 1:** *hopper*^*Bd-*we^ element transformation experiments in *D. melanogaster* and *A. suspensa*

Host^a^	Vector	G_0_ eggs injected	G_0_s mated^b^	No. G_0_ lines	No. fertile G_0_ lines	No. G_1_ transformant lines	Transformation frequency^c^
*Dm*	pKhop [*Dmwhite*+]	436	131	98	79	2	0.025
*Dm*	phop [*PUb-DsRed.T3*]	1056	203	170	129	2	0.016
*As*	phop [*PUb-DsRed.T3*]	1453	389	103	94	9	0.096

^a^*Dm*: *D. melanogaster w*[m] strain; *As*: *A. suspensa* wild type strain

^b^total number of G_0_ adults mated based on 70, 140, and 80 single G_0_ adult matings (to 3 opposite sex adults) for *D. melanogaster* pKhop[*Dmwhite*^+^] and phop[*PUb-DsRed.T3*] transformations, and the *A. suspensa* phop[*PUb-DsRed.T3*] transformation, respectively; additional G_0_s were mated in groups of 2–3 same sex G_0_ adults to opposite sex adults

^c^transformation frequency calculated as number of transformant lines per number of fertile lines

The second *hopper* transformation was similarly performed in the *Drosophila w*[m] strain, but using the phop[*PUbDsRed.T3*] vector, marked with a strongly expressing fluorescent DsRed variant [18]. For this experiment 1056 eggs were injected, from which 203 G_0_ adults survived that were backcrossed to *w*[m] in 170 matings (Table 1). From 129 fertile G_0_ matings, G_1_ transformant progeny expressing full-body DsRed fluorescence from 2 independent matings (F73A and F74A) were observed (Fig. 1b), yielding a transformation frequency of 1.6% per fertile G_0_. Similar to the F11A line, F74A yielded four sib transformants that also exhibited female-specific marker expression that was X-linked.

The third *hopper* transformation also tested the phop[*PUbDsRed.T3*] vector, but in the Caribbean fruit fly, *A. suspensa*, that is in the same Tephritidae family as *B. dorsalis*. In this experiment 1453 eggs were injected from which 389 adults survived that were backcrossed to wild type *A*. *suspensa* in 80 individual G_0_ matings and 23 group matings (Table 1). From 94 fertile G_0_ matings, G_1_ DsRed transformant progeny from 9 independent matings (lines F2M, F5M, F7, F30, F36, M8A, M31, M32, and M35) were observed (Fig. 1c), yielding a transformation frequency of 9.6% per fertile G_0_. All of the lines, except for F5M, expressed DsRed in both male and female adults that presumably resulted from autosomal integrations. Fluorescent marker expression in F5M, however, was only observed in male progeny that would imply a Y-linked insertion.

### Verification and molecular analysis of genomic *hopper* transformant integrations

**Fig. 2:**
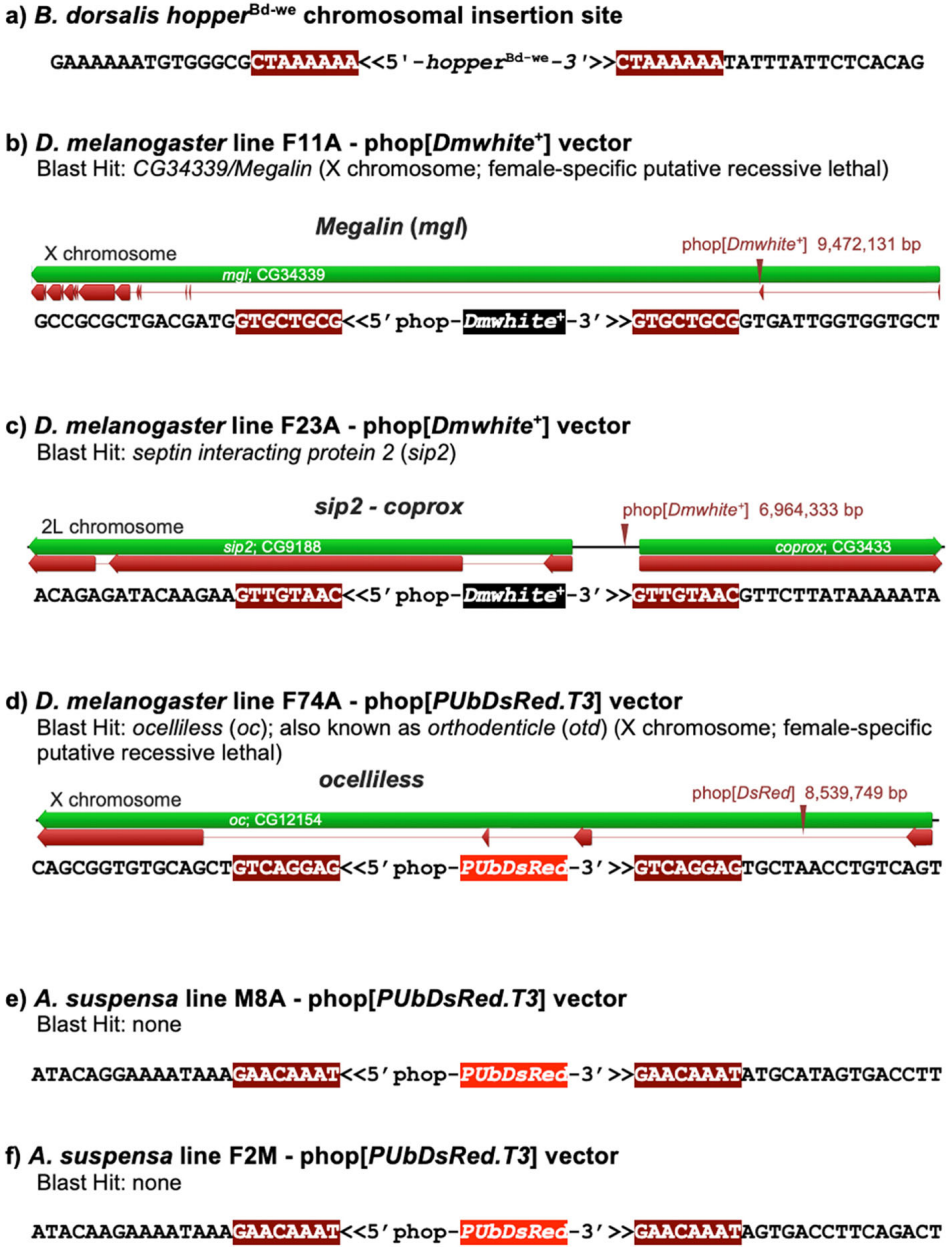
Flanking genomic insertion site sequences for the *B. dorsalis hopper*^Bd-we^ transposable element in the *B. dorsalis white eye* strain genome (**a**) compared to pKhop [*Dmwhite*^+^] and phop[*PUbDsRed.T3*] vector insertions in *D. melanogaster* (**b**-**d**) and *A. suspensa* (**e**-**f**). Twenty-three (23) nucleotide 5′ and 3′ flanking genomic sequences shown for vector insertions in the designated transformant lines, including the proximal 8-bp duplicated insertion site sequence (brown outline). The highest identity Blastn hit (> 95%) for the complete vector insertion site sequences is provided (see Additional file 1: Fig. S1 for complete insertion site sequences). Below flanking genomic sequences (**b**-**d**) is a schematic diagram of the genomic insertion site position (annotations: green, gene; red, exons; arrow, insertion site position)

To verify that transformant lines from each experiment were the result of genomic germline transposon-mediated vector integrations, 5′ and 3′ sequences flanking the *hopper* vector were isolated by TAIL-PCR or inverse PCR and sequenced (Fig. 2 and Additional file 1 Figure S1). Transposon-mediated vector genomic integrations were indicated by intact terminal sequences of the *hopper* vector, but having duplicated 8-bp insertion sites and flanking sequences that differed from *hopper*^Bd-we^ and its insertion site sequences in the vector plasmid (Fig. 2a), eliminating the possibility of vector integration by a recombinant event. Additional evidence was provided for some transformations whose genomic insertion sites could be identified and mapped by BLASTn database searches.

For the *D. melanogaster* pKhop[*Dmwhite*^+^] F11A transformant line, TAIL-PCR yielded 221-bp for the 5′ flanking sequence and 151-bp for the 3′ flanking sequence having nearly 98% identity with the X chromosome-linked *CG34339/Megalin* 141 kb gene from a BLASTn search of the *D. melanogaster* NCBI database (Fig. 2b and Additional file 1 Figure S1a). The 8-bp duplicated insertion site sequence was GTGCTGCG compared to the *hopper* vector CTAAAAAA duplicated insertion site isolated from the *B. dorsalis white eye* strain genome. Notably, mutant-rescue transformants expressing red eye pigmentation were limited to females suggesting that the vector insertion created an X-linked recessive lethal mutation, that was supported by a backcross of F11A females to *w* [m] males. This resulted in 111 F_1_ white-eye males and 203 females that were 48% red eye that is consistent with an X-linked recessive lethal based upon male/female survival, marker phenotype segregation, and with previously created X-linked mutations in the *Megalin* locus [19].

For the *D. melanogaster* pKhop[*Dmwhite*^+^] F23A transformant line, TAIL-PCR yielded 328-bp for the 5′ flanking sequence and 884-bp for the 3′ flanking sequence. A *D. melanogaster* BLASTn search showed that this sequence has greater than 98% identity to the chromosome 2 intergenic region between the *septin interacting protein 2* (*sip2*) and *coproporphyrinogen oxidase* (*coprox*) genes at the 27C7 locus (Fig. 2c and Additional file 1 Figure S1b). The 8-bp duplicated insertion site sequence was GTTGTAAC.

For the *D. melanogaster* phop[*PUbDsRed.T3*] F74A transformant line, TAIL-PCR yielded 1017-bp for the 5′ flanking sequence and 102-bp for the 3′ flanking sequence. A *D. melanogaster* BLASTn search showed that this sequence has nearly 97% identity to the first intron of the X chromosome-linked *ocelliless* (*oc*) gene at locus 7F10-8A1 (Fig. 2d and Additional file 1 Figure S1c). The 8-bp duplicated insertion site sequence was GTCAGGAG. An interesting aspect of this *hopper* integration is that, similar to the F11A line, transformants expressing DsRed fluorescence were limited to females consistent with the creation of an X-linked recessive lethal mutation. Segregation analysis of a backcross of F74A females to *w*[m] males resulted in 76 non-fluorescent F_1_ males and 140 females that were 42% DsRed fluorescent consistent with an X-linked recessive lethal based upon male/female survival and marker phenotype segregation. However, while *oc* mutations cause neural defects that result in the absence of the ocelli photoreceptors in homozygous mutants, homozygotes remain recessive viable, though an allele of the polycistronic *oc* locus, *orthodenticle* (*otd*), has a moderate dominant phenotype and is recessive lethal [20]. Thus, the vector insertion appears to be more highly related to the *otd* phenotype rather than *oc*. Another consideration for this insertional mutation is that it occurs in the *oc* first intron, that would typically be considered silent, though an *otd* enhancer has been identified in intron 3 [21] and conceivably another *otd* enhancer or regulatory region might exist in intron 1 for this complex locus.

For the *A. suspensa* phop[*PUbDsRed.T3*] M8A transformant line, TAIL-PCR yielded 946-bp for the 5′ flanking sequence and 262-bp for the 3′ flanking sequence (Fig. 2e and Additional file 1 Figure S1d), but was inconclusive for the other *Anastrepha* transformant lines. However, inverse PCR of line F2M yielded genomic sequences of 73-bp at the 5′ junction and 861-bp at the 3′ junction (Fig. 2f and Additional file 1 Figure S1e). A high quality assembly for the *A. suspensa* genome is currently unavailable, and a BLASTn search of the NCBI nr/nt database, as well as the *D. melanogaster* and *Ceratitis capitata* (medfly) databases, found no significant identities for the genomic insertion site of either transformant line. However, the two insertion site sequences did share the same 8-bp duplicated GAACAAAT insertion site sequence, and there was high identity between the adjacent 37-bp sequences at the 5′ junction. Considering the highly repetitive A/T content for both insertion sites (Additional file 1 Figure S1d,e), 83% for M8A and 66% for F2M, it may be assumed that the insertions occurred in species-specific non-coding regions, and highly repetitive regions may have hindered the PCR isolation of insertion sites for the other lines.

## Discussion

The *hopper*^Bd-we^ transposable element previously discovered in the tephritid fruit fly, *B. dorsalis*, has been shown to function as a transposon vector for germline transformation in two other dipteran species, consistent with it being an autonomously functional element. Relatively modest 1.6 and 2.5% transformation frequencies were achieved with two independently marked vectors in *D. melanogaster*, using mutant-rescue restoration of eye pigmentation as a marker for one vector, and ectopic DsRed fluorescent protein expression as marker for another. The latter vector also achieved transformation in the tephritid fruit fly, *A. suspensa*, which is more closely related to *B. dorsalis*, at a higher rate of 9.6% per fertile G_0_, however additional experiments in these and related dipteran species will be necessary to determine if there is a significant species-specific relationship to transpositional frequency. Though not shown to be a highly robust vector in this initial study, *hopper* yielded transformants in each individual experiment, and most of the transformant lines created in this study have been cultured in the laboratory for a minimum of 6 to 8 years. Thus, *hopper* appears to be a reliable and stable vector for these two species, but as with all newly discovered vectors, actual transformation efficacy and range of function will depend upon continued tests in insects from several Orders. Since the functional element exists in *B. dorsalis*, and a closely related (if not the same) *hopper* element exists in the closely related species, *Z. cucurbitae* [14], its ability to routinely create stable transformants in these and related tephritids also remains to be evaluated.

Beyond the possibility for differential transpositional activity in the two species, the limited number of insertions molecularly characterized, nevertheless, raise the possibility for preferential insertion sites. For *D. melanogaster*, the three transformant lines analyzed resulted from vector insertions within actively expressing genes (including exons and an intron), or within a short intergenic sequence. In contrast, both *hopper* vector insertions in *A. suspensa* were localized to highly repetitive A/T enriched non-coding regions. Furthermore, both insertions targeted identical 8-bp GAACAAAT genomic sites having an additional 37-bp sequence identity at the 5′ junction. Transposon vector insertions in the same variable target site sequence do occur, if not the same genomic target site, but these have usually been discovered in large screens of a re-mobilized transposon [22, 23]. In addition, two of the four *Drosophila* transformations were putative X-linked recessive lethal insertions, and one of the nine *Anastrepha* transformations was a putative Y-linked insertion. While sex-linked insertions are occasionally found in transformations with other vectors in various species, we are not aware of a bias for this type of insertion site preference. Together, the findings from this initial limited study of *hopper* transpositions are intriguing that will require an expanded analysis for verification.

One of the benefits of transposon-mediated transformation not afforded by other types genetic manipulation is their ability to randomly insert into the genome that allows transposons to create mutations by insertional mutagenesis, or be used as ‘traps’ to identify and isolate enhancers [24], proteins/exons [25, 26] and promoters [23]. These methods are of particular interest in species where newly identified genetic reagents, such as a variety of promoters and genes affecting viability and sex determination, are essential for genetic modifications that improve population control systems such as SIT. However, these transposon-based tools typically rely on the post-integration remobilization of the requisite vector for each system, and not all transposons are subject to remobilization in all species, possibly due to species-specific transpositional silencing. Most notable for this effect is the yellow fever mosquito, *Aedes aegypti*, that has been transformed with several vectors that have been completely, or highly, refractory to re-mobilization [27, 28]. Thus, new transposon vectors increase the possibility for developing more effective re-mobilization systems, and also increases the means to create secondary transformations in transgenic strains without re-mobilizing the primary integration.

For more straightforward genomic insertions of gene constructs for comparative gene expression and other functional studies, and strain modification for pest biological control or enhanced fitness for beneficial insects [29], transposon-mediated transformation is, currently, a more efficient if not more reliable process than the use of CRISPR/Cas9-mediated integration. Gene-editing based insertions, depending on homology-directed repair donor templates, are generally size-limited to a few thousand base pairs [2], while many constructs for strain modification are 10–15 kb in length, with off-target modifications most easily minimized in species having a high quality whole genome sequence [30]. In addition, while efficient DNA delivery remains an impediment to germline transformation for many species, this process can also be a limitation for CRISPR/Cas9 modification, and it is notable that development of several CRISPR/Cas9-mediated gene drive systems rely on transposon-mediated transformation to create ‘helper’ strains with a genomic source of Cas enzyme [31].

Beyond the advantages that a new functional transposon vector may have for functional analysis and transgenic strain modification, it is also recognized that functional Class II transposons are agents of evolutionary change resulting from insertional mutagenesis [32], and a comparison of variations in non-functional defective elements in closely and distantly related species provides insights into their phylogenetic relationship [33]. Indeed, we already know that *hopper* is currently the most distantly related insect *hAT* element to *D. melanogaster hobo*, being equidistant to *Activator* in maize, and that elements closely related to *hopper*^Bd-Kah^ exist in the *Bactrocera* species, *Z. cucurbitae* [13]. Thus, a more extensive survey of *hopper* elements in *B. dorsalis* and *Z. cucurbitae*, and tephritid species in general, may contribute to resolving their evolutionary relationships [34], and better define the limits for the practical use of *hopper* vectors for genetic modification.

## Conclusions

The previously isolated *hopper*^Bd-we^ transposable element was verified as an autonomous functional element by its ability to mediate genomic transpositions in the germline of two dipteran species. This is the second insect *hAT* element, in addition to *Hermes*, known to be functional in a non-drosophilid species thereby expanding the tools available for genetic modification and genomic functional analysis in these insect species, and possibly others. The discovery of both a degenerate defective and functional *hopper* element in *B. dorsalis*, that might also exist in *Z. cucurbitae*, suggests that this distantly related *hAT* element has had a long history in the *Bactrocera* genus and may be instrumental in clarifying its phylogenetic complexity.

## Methods

### Insect rearing

The *Drosophila melanogaster w*[m] strain and transformant lines were maintained at 25 °C and reared under standard laboratory conditions [35, 36]. An inbred wild type colony of *Anastrepha suspensa* (Homestead, Florida) and transformant lines were also reared under standard laboratory conditions at 27 °C and 60% humidity on a 12 h light:12 h dark cycle [37].

### Vector and helper plasmids

pUChsHopper. The *hsp70*-regulated *hopper*^Bd-we^ transposase helper plasmid, pUChsHopper, was created by first isolating the *hopper*^Bd-we^ transposase coding region and polyA termination sequence as a blunt-*Eco*RV/*Xba*I fragment from pBS*hopper*^Bd-we^ [14]. The *hopper*^Bd-we^ fragment was then ligated into a *Bam*HI-blunted/*Xba*I site of pUChsRB, downstream of the 457-bp *Xba*I-*Xmn*I 5′ non-translated sequence from *D. melanogaster hsp70* [17].

pKhop[*Dmwhite*^+^]. The pKhop[*Dmwhite*^+^] vector was created by isolating a 4.2-kb *Eco*RI-blunted fragment of the *D. melanogaster* mini-*white*^+^ cDNA gene [38] and ligating it into the pK*hopper*^Bd-we^ blunt-*Hinc*II site at nt 922 within the open reading frame, thereby eliminating functional transposase.

phop[*PUbDsRed.T3*]. The *hopper*^Bd-we^ vector marked with *D. melanogaster polyubiquitin* (*PUb*)-regulated DsRed.T3 [18] was created by isolating *PUbDsRed.T3* from pBXLII [*PUbDsRed.T3*] as a blunt-*Sma*I /*Sac*II 3.0-kb fragment ligated into the blunt- *Sna*BI/*Sac*II deletion in pBS*hopper*^Bd-we^ [14].

### Transformation experiments

Pre-blastoderm embryo injections for germline transformation were performed as described for *D. melanogaster* [36] and *A. suspensa* [37], but using DNA mixtures having vector/helper concentrations in injection buffer (5 mM KCl; 0.1 mM sodium phosphate pH 6.8) of of 400/200 ng/μl for *Drosophila* and 600/400 ng/μl for *Anastrepha*. Dechorionated embryos were injected under Halocarbon 700 oil, placed within an oxygenated chamber for 16 to 20 h and subjected to a 37 °C heat shock for 45 min, and then reared at 25 °C until adult eclosion. Eclosed G_0_ adults were mated either individually to two or three *w*[m] adults, or in groups of two to three G_0_ males or females backcrossed to *w*[m]. G_1_ eggs were collected for 10 to 14 days and reared under standard conditions. Putative transformant G_1_ adults were screened for either eye pigmentation under bright field for pKhop[*Dmwhite*^+^] injected flies, or whole body (though primarily thoracic flight muscle) DsRed fluorescence for phop[*PUbDsRed.T3*] injected flies. DsRed epifluorescence was observed under a Leica MZ FLIII microscope using a Texas Red filter set (ex: 560/40; em: 610 LP; Chroma).

### Isolation of transgene integration flanking site sequences

Flanking genomic sequences of *Dm_*F11A, *Dm_*F23A, *Dm_*F74A and *As_*M8A transgene integrations were isolated by TAIL PCR as described by Liu and Whittier [39], starting with 300 ng of genomic DNA in the primary PCR reaction. The Platinum Taq Polymerase (Thermo Fisher) was used for all PCR reactions in a total volume of 25 μl per reaction. Oligos used for the isolation of the 5′ *hopper* vector insertion-site flanking sequences by TAIL PCR were the degenerate primer AD3 (AGWGNAGWANCAWAGG) and the *hopper*-specific primers R1_5hop_P952 (ACATTTGCTGAATATAATACCATTTACTTG), R2_5hop_P953 (GATATCTACTTGCATAAAATCATTCATTCG), and R3_5hop_P954 (ACTATCGAATGAATGAAAATTGCTGAAC). Oligos for the isolation of 3′ *hopper* flanking sequence were the degenerate primer AD3 (AGWGNAGWANCAWAGG) and the specific primers F1_3hop_P955 (ACCTCGATATACAGACCGATAAAACACATGC), F2_3hop_P957 (CGTACGTCACAATATGATTATCTTTCTAGG), and F3_3hop_P958 (TCATTCAGTCATTAACAATCGATAGTTG). For the *As*_F2M transgene integration, the flanking genomic sequences were isolated by inverse PCR using *Hae*III to digest the 5′ junction and *Msp*I to digest the 3′ junction. The circularized insertion sites were then isolated and sequenced using the primers P967_For (TGAAATTAAGCAGGTTGGCAACTTG) and P952_Rev (ACATTTGCTGAATATAATACCATTTACTTG) for the 5′ junction, and P969_For (CGTTGTGACAAATAGTTTTTGCTTCC) and P956_Rev (TTGTTGTTTGAAGAGCACGCCTTTGC) for the 3′ junction.

## Supplementary Information

**Additional file 1.**

## Data Availability

The datasets, sequencing data and materials supporting the conclusions of this article are described within the article and its additional file, and materials will be provided by contacting the corresponding author.

## Abbreviations

A/T: Adenosine/Thymidine
*Bd*-Kah: *Bactrocera dorsalis*-Kahuku
*Bd-we*: *Bactrocera dorsalis*-*white eye*
BLASTn: Basic Local Alignment Search Tool nucleotide
*coprox*: *coproporphyrinogen oxidase*
CRISPR/Cas9: Clustered Regularly Interspaced Short Palindromic Repeats/CRISPR-associated 9 gene
*hAT*: *hobo, Activator, Tam3*
*hsp70*: *D. melanogaster heat shock protein 70*
NCBI: National Center for Biotechnology Information
*oc*: *ocelliless*
*otd*: *orthodenticle*
PCR: Polymerase chain reaction
phop [*PUbDsRed.T3*]: Plasmid hopper [*polyubiquitin-DsRed.T3*]
pKhop [*Dmwhite*^+^]: Plasmid kanamycin-resistant hopper [*D. melanogaster white*^*+*^]
*sip2*: *septin interacting protein 2*
TAIL-PCR: Thermal asymmetric interlaced PCR
TIR: Terminal inverted repeat
