# Supplementary material for: The hAT-family transposable element, hopper, from Bactrocera dorsalis is a functional vector for insect germline transformation
Source: BMC Genet. 2020 Dec 18;21(Suppl 2):137. doi: 10.1186/s12863-020-00942-3 (PMC7747358; doi:10.1186/s12863-020-00942-3)
Supplement: Supplementary file 1 — Additional file 1. [file 12863_2020_942_MOESM1_ESM.docx]

**Additional file 1:**

**Supplementary Figure S1.** Flanking genomic non-degenerate insertion site sequences for the pKhop[*Dmwhite*^+^] and phop[*PUbDsRed.T3*] vector insertions in *D. melanogaster* (a-c) and *A. suspensa* (d, e), that were generated by TAIL PCR (a-d) and inverse PCR (e). The highest identity (>95 %) Blastn hit for the complete insertion site sequence is provided, or none for no high identity hit (note: an annotated genome for *A. suspensa* has not been published). Duplicated 8-bp genomic insertion site sequences flanking the 5’ and 3’ terminal vector sequences are outlined in brown.

**a) *D. melanogaster* F11A - phop[*Dmwhite*^+^]**

Blastn hit: *CG34339/Megalin* (X chromosome; female-specific putative recessive lethal); Vector insertion localized to an exon in the *CG34339/Megalin* gene at position 9,472k on chromosome X; Heterozygous DmF11A females backcrossed to *w*[m] males: 111 males (0 yellow eye); 203 (97 fluorescent)

TTTAAGTCAATTTTTTGGCTTAGCTTTTAAAGTCAATTAACATTTATAGCAATATTTGTGGGTATATTTGGGAATTTAGCTCGACTCACCTGGTGATCCATGACGGTTGCGCGGTCCTTCGGTGCGCACGGCGAGCAGGGTTTCCAGGTTGCTGATGGCCACAAGGGCCAGTAGCAGCAGCGCCAGGGTTGTCAACTGGCCGCGCTGACGATGGTGCTGCG**<<5’phop-*Dmwhite*^+^-3’>>**GTGCTGCGGTGATTGGTGGTGCTGCTGCTGCTGCAG CCTCCGATCGATGGCGCTGCTGCTGTGATGCTGTGAATGGCGGTCGCCGATGGGCGAGGATGATGAAGATGATGGTGGGCCCGATGGGGCGGCATCAGTGACCTGCAGCGGATCG

**b) *D. melanogaster* F23A - phop[*Dmwhite*^+^]**

Blastn hit: *sip2 – coprox;* Vector insertion localized to the chromosome 2L intergenic region between *sip2* and *coprox*:

ATCTTCTTAAACCGTGGGTCTAAGAAGGTCGATTTGGCACACATCTTGTCAGACTCTGGTTGGCCCCCTGGTCCGGCGCACCAGTTGAAAGGTGCCCAGCGATACCAGGGATATCGTGTGACGGAGTGCGTTCATTATGTGCTCTCGCCTACTTCAAATTTTTAACTTAACTTAAGTACTTTCGCCGAACGCAGTTTGTTTGTTATCTTTCACTGACCCGGCATTTCTAGTTGCATAGTCGCCGGTGTGACCGTGATATCACCAAAATATACCGAAAATAGGGGGCTCGCGATTTTGTTCTATTTACAGAGATACAAGAAGTTGTAAC**<<5’phop-*Dmwhite*^+^-3’>>** GTTGTAACGTTCTTATAAAAATATTATATATTGTTAAGAACCCTAGCTAGCTCAAT ATGTCTGTATAT ATACACAAAATAATAATAATAAATAATATATGTTAAAAGTTACGGAGCAGTACAATTGCTTTTAATGGTAGACATATTTGTCTTAATTTTATATTATTATTATAAGTGATATAACTCCGATGATGTATATGTGTAGTGTCCATTGGAATACCCCATTCAAAAATATAAGCGCAATACGGGCACTCTGTCGTGGCATTTGAAATAAACGACTGCAAAGGCGCGTCGATCGTTGGCAAATGAGCGTGCTGTGAAGAAATCCATGAAATATTTGCGTGTGCGCCTTGAAGATTAAGGAAGATCTGCGTGAATACTATTGCCAGAAGGTAGGTGATCGACTGGAGCGAACTCTAAATCCTCTGAACTATAGTCAACGCGCAATTATACGTGTTTATCCCGTCGACTGGAGTTTTGCACCCAGGACACACGCACACTCGCACACTCACGATGTCTGGCCTCAAGAAATTCCAAAACACTGAGGAATGGCGTCAGAAACTCCGTCAGTTTCTAATGACCGAGCGGTAAATACACATTGAAACCCATCCTTGGATAGTGTTGCACTAGTGTTCGATGAATTTGTAGTCATTTACTTTCAAACAAGCCACCTGCATGTCAAAATTGTTAGAGAAACAGTACGCATAAATAGTTTGGATAGTTTAGACACACATACAATTTAGTTGAGAAAACTAATAACAGTTTTCCTTTTTCAGCTTGCCGAAAAAGCCCGTGAAAATGGACGAAACGAANNATATGCGCTATAGTTTGGAGATCGACGAGGATTTGAGGAGACCCTTGGNGNNNCGGGNCAAGAAAGTTCTG

**c) *D. melanogaster* F74A - phop[*PUbDsRed.T3*]**

Blastn hit: *oc*/*otd* (X chromosome; female-specific - putative recessive lethal); Heterozygous DmF74A females backcrossed to *w*[m] males: 76 males (12 fluorescent); 140 (58 fluorescent);

Vector insertion localized to *ocelliless*/*orthodenticle* (*oc*/*otd*) intron 1:

TGGGTCCGAGAAGAAGAAGAAAAAAGAAAAAACCTCGTCGTCAAACTCTCAGCATCNNGTCAAAAAAAAACCGAANGTGACACATTTTCGAAAACATCCCGGCAAAGAAANGGGTTGGAATATGGAATGAAANGGCGATCTACGGCACTACGTGATTTTCTAGACATTTCACAGCCCCCTTTGGCCTTCCGCCCTCGCTGATTATGAAAAATCATGGTAAATGGCAAAAAGAGCAAAATAAAAAATAAAAAAAAAAAAGAAAACAAGAAGGCCCCGCTGGCATTTTTTTCATCTTTTTTTTTTTTGAAGAGATCCATTGTGAGGCCGAAACGAGTCGAGACAAAATCATTTGGGCTGGACATATATCACGTACGAACGGCGGACACTTTCATTATAAATTCCATTCGGCATTTTCATGATTTTCGGTTCGGTACGATCTTCTGATTTGCTCCATTCTCAGCGAATGTTTTTCAATTTTCACTCTCGATTTCTATATCTACTCCCATTTCCGCCGCCTCACAAACATTTCAATTCGTTTGTCCGCGCGTAATTACGGTCAAAATAATATTTATTGGACAAACAGCCGGGAGGAAAACAAAATAAAGCCCAGAGATTCAGCGATGCTACCGTAAGATTTTGGGGGTTTTTAGAGATTTTCTAGAGTGCTGTAATGGTGAGAGGGGGGGGGGGATGGGGTACTGAACCCTTTTTAAGCCCGCACATCCAAGTGTTTTGGTTCGCTGATGATCCGACTGACGGTCCCTGGGAAAGTCGAAGAGTTAAAGATTCGAATAGAAATCGAACTGCCACTTGGCTACTGTTCATTTGGGCCATGGGAATTGTGGATCTCCACGCCGTTTGCCCGTTTAATTAATGGTCCGTCTTTGCATAACTTTCGGTCTACGCATTTGGCTCTGACTAATATGAAATATTAAATGCCGTCTCGGCGATCGCATATTTATATCTATATTTAAATTTAGTCNAGTCGGGCATACAGCGGTGTGCAGCTGTCAGGAG**<<5’phop-*PUbDsRed*-3’hop>>**GTCAGGA GTGCTAACCTGTCAGTCGCCGGATCGCAGTTAGTATTAGTAGTAGTAGTAATCGCTGCTAGTTTCTGGTAGTCGCTGCTATTAGCCGCCTTAACA

**d) *A. suspensa* M8A - phop[*PUbDsRed.T3*]**

BLASTn hit: none

TTTTGCAGGTTACCTTTAAAGTTATTGAAGATATTAAAAGAGCGCTCTTAAATATAAAAAAATAAAATAAATAAATAATAAAAAAAAATATTAAATAAAATTAAAAAAATAAAATTAAAAATTAAATAAAATTAAAACAAAAATGTTATTCAAAAACAATGCAAATAAAAAATAAATAAATAAAAAATTAAATAAAATTAAAACAAAAATGTTATTAAAAAATCAATACAAATTAAAAAAAAAATATATAAAAATATGTAAATAAACAAACAAAAATAAAAGAAAAAAACATAAATATATGAAGAAATAAATAAAACGAAATAATTAAAAAAAATCTCAAACAAAAAAAAAAAATAAAAAAAAATTTAAAAACAAAATAAAATAATTAAAAAATAAAATAAAAAATAAATTTAAAAACAAAATAAAATAATTTAAAAAAAATAAAATAATTACAAAAAATAAAATAATTAAAAAAATGTTATTAAAACAATACAAATAAATACAAATAATAAAATAAAAATATGAATTAAAAAAGTATGTAAATAAAAAATAAATAAAAAGGGCATAATTAAAAAATATATATATAAATTAGAAAGAAATAATTAAAAAAATTATCAGCAAAATAAATAAATAAAAAAATAGTTTAAAAATAATAATAATAAACAAACGAAAATTATAATAATTAAAAAACAAACAATAAATGCTTTGTGGACGCAAAATACAGAGAATATTTTTTTCAAACCATCCTTTTTAATAATCTATTTCATTGCTATAAACAAAGAATACGTATAATAAAATAAAACAGAAAAAATTCAATGTGTTTCGCGTTCGAATTCTTATACCCGCAACTGTAGAATAGAAATAGAACCAGCCGACACTTATCACAATTATTGATTATTTGGAGTGCACTTTTTCAACAGATAATACAGGAAAATAAAGAACAAAT**<<5’phop-*PUbDsRed*-3’hop>>**GAACAAAT ATGCATAGTGACCTTCAGACTCTCAAATTTTTAATTCTGCTATAATATAGAGGCAGATACATTGTTTCCAAGCAAAAAATGTACTATAATAATATATTTAATATCATATATTTATACTTTTTTTTGTATTTCGAAGAATAAATTCTGACAACAGCCAAAAATTTAATTAATATGCACGAAATAATGGCTCAACTATGTTGTTTGTTGTTGCAATAGCATAGGAAACTGTTTATTGTTGTTGAAAAAAACCGCAT

**e) *A. suspensa* line F2M - phop[*PUbDsRed.T3*] vector**

Blast hit: none

GACACCCGTAACGATTACTGATTCTTGTGAGTGCACTTTTTCAACAGATAATACAAGAAAATAAAGAACAAAT**<<5’phop-*PUbDsRed*-3’hop>>**GAACAAATATGCATAGTGACCTTCAGACTCTCAAATTTTT AATTCTGCTATAATATAGAGGCAAATACATTGTTTCCAAGCAAAAAATGTACTATAATAATATATTTAATATCATATATTTATACTTTTTTTTGTATTTCGAAGAATAAATTCTGACAACAGCCAAAAATTTAATTAATATGCACGAAATAATGGCTCAACTATGTTGTTTGTTGTTGCAATAGCATAGGAAACTGTTTATTGTTGTTGAAAAAAACCGCATGGCCAATGAACAAAAATATTTAAACATTGGTTTATTTTTTTAAGGCTCACATTATATTAGTTGAAGTTTTGAATGCATACCGCGGAGCCTTTCTAGTGCCAAATCTCAAGATGTGAATTAGTACATTTCGTCGGATCCTGCTATACGGAAATTTAAAGGAGAAATTACGTGTCACGAGTTTTGTGTTTCGTGAAGTTAGTTGTGACAATATAACATTGGGCACATTTCTCTATACCATCACGACTAAGGTGAAAAGTAGGAACAGGCAGCCGACAAAATGTATGGTGTTTATGGACACAGTTCACTATCGAGAGCAGCGGAAAAGCGTGATTTCAACGACTCCATTTCTGTGCTGTGGACTCCTATGTTGCCTCACAAATAAAAGCACCTTTGAACTTTTATTAGAAATTCATTTCATTTCAGAAAAATACTCGGCAATTTAAAATTGTCAACTGAAGTGTGACCGAACCGAAACCATTGGGCATCCAATACAGATCTTACTGTAAATGTGTCATTCACAAACGTGCTCGGCAACTATGTATGCTGGTCGAACTGCATATTCTAATGAAAATTTGTACTTATTTGTATACACCATGAAA
